# Using the social vulnerability index to assess COVID-19 vaccine uptake in Louisiana

**DOI:** 10.1007/s10708-022-10802-5

**Published:** 2022-12-09

**Authors:** Mohammad Alfrad Nobel Bhuiyan, Terry C Davis, Connie L Arnold, Nasim Motayar, Md. Shenuarin Bhuiyan, Deborah G Smith, Kevin S Murnane, Kenneth Densmore, Maarten van Diest, Steven R Bailey, Christopher G Kevil

**Affiliations:** 1 Department of Medicine, Louisiana State University Health, Shreveport, LA USA; 2 Department of Medicine, Department of Pediatrics, and Feist-Weiller Cancer Center, Louisiana State University Health, Shreveport, LA USA; 3 Department of Pathology and Translational Pathobiology, Louisiana State University Health, Shreveport, LA USA; 4 Department of Pharmacology, Toxicology and Neuroscience, Louisiana State University Health, Shreveport, LA USA; 5 Center of Excellence for Emerging Viral Threats, Louisiana State University Health, Shreveport, LA USA

**Keywords:** Social vulnerability, COVID-19 vaccination, Spatial analysis, Social determinants of health

## Abstract

Using data from the Louisiana Department of Public Health, we explored the spatial relationships between the Social Vulnerability Index (SVI) and COVID-19-related vaccination and mortality rates. Publicly available COVID-19 vaccination and mortality data accrued from December 2020 to October 2021 was downloaded from the Louisiana Department of Health website and merged with the SVI data; geospatial analysis was then performed to identify the spatial association between the SVI and vaccine uptake and mortality rate. Bivariate Moran’s I analysis revealed significant clustering of high SVI ranking with low COVID-19 vaccination rates (1.00, *p* < 0.001) and high smoothed mortality rates (0.61, *p* < 0.001). Regression revealed that for each 10% increase in SVI ranking, COVID-19 vaccination rates decreased by 3.02-fold (95% CI = 3.73–2.30), and mortality rates increased by a factor of 1.19 (95% CI = 0.99–1.43). SVI values are spatially linked and significantly associated with Louisiana’s COVID-19-related vaccination and mortality rates. We also found that vaccination uptake was higher in whites than in blacks. These findings can help identify regions with low vaccination rates and high mortality, enabling the necessary steps to increase vaccination rates in disadvantaged neighborhoods.

## Introduction

In a comparatively short time, the COVID-19 pandemic has caused global devastation and claimed 4.98 million lives (Global Cases and Vaccines, 2021). In March 2020, Louisiana became, and has remained, a hotspot for COVID infection (Louisiana Office of the Governor, 2020). According to the census data, Louisiana’s population is 32.8% Black and 62.8% white (United States Census Bureau, 2021). With respect to vaccination against COVID-19, 35.9% of Blacks and 62.6% of whites in Louisiana have been vaccinated (Louisiana Office of the Governor, 2020). A similar racial disparity has been found throughout the United States (Gaynor and Wilson, 2020; Scott et al., 2022; Centers for Disease Control & Prevention report, 2020; Taylor et al., 2022).

Medical research on chronic health conditions has increasingly focused on retrospectively identifying risk factors that may predict severe disease. Racial disparities have also come to light during this research, with Blacks at a higher risk from chronic conditions (Chowkwanyun et al.,^2020^; Selden et al., 2020). However, less effort has been applied to using existing socioeconomic tools to predict health outcomes. Had such tools been used during the beginning of the COVID-19 pandemic, preventive strategies could have been developed and used in more vulnerable communities, decreasing the morbidity and mortality in these at-risk areas. Recently, the Distressed Community Index (DCI) (Hawkins et al., 2020), Neighborhood Deprivation Index (NDI), and Area Deprivation Index (Area Deprivation Index, 2019; KC et al., 2020) have been evaluated for use in determining the impact of COVID-19 on at-risk socioeconomic communities. Hawkins et al., evaluated the DCI and found that communities with lower education rates and primarily black communities had more cases and higher fatalities from COVID-19 (Hawkins et al., 2020). Madhav et al., (2020) identified disadvantaged neighborhoods using the NDI and found their residents to have a 40% higher risk of COVID-19 transmission.

The Geospatial Research, Analysis, and Services Program (GRASP) of the Centers for Disease Control and Prevention (CDC) has created a database ranking of communities based on social variables, assessing their risk for worse outcomes during public emergencies (Agency for Toxic Substances & Disease Registry, 2018). The Social Vulnerability Index (SVI) is a tool that examines the impact of external stressors, such as socioeconomic status, household composition, minority status, and access to housing and transportation, on public health outcomes. The data generated using the SVI has been used to map the communities most likely to need support in emergencies (Cutter et al., 2003; Palaiologou et al., 2019). All countries, including the United States, included experience changes in population, socioeconomic conditions, and overall development that affect the SVI. In a discussion of the spatial and temporal trends of the SVI, Cutter et al., (2010) showed that regional variability caused the SVI to increase over time. In numerous studies, the researchers linked SVI with socioeconomic behavior (Amaro et al., 2021; Reis et al., 2017), health outcomes (Khan et al., 2021), and climate-sensitive hazards (Cutter et al., 2010; Emrich and cutter, 2011; Emrich et al., 2020). Recently, the CDC evaluated its use in COVID-19 vaccination coverage in the United States (Hughes et al., 2021). They found higher vaccination coverage in lower SVI regions. They suggested that vaccination coverage be monitored using SVI metrics to focus public health interventions on achieving equitable coverage for the COVID-19 vaccine (Hughes et al., 2021). In the beginning stages of the COVID-19 pandemic, Louisiana had the highest number of reported cases and deaths. During the time this study was conducted, Louisiana had the fifth-highest number of reported deaths and one of the lowest vaccination rates in the country, ranking seventh overall (Madhav et al., 2020). The SVI could have allowed early identification of communities at risk for mortality and enabled local public health officials and healthcare workers to focus on preventive strategies to reduce cases and fatalities in these areas. Our study aimed to evaluate the utility of SVI in identifying at-risk regions to allow public health departments to target resources and communications to improve vaccination rates in those areas. We used statistical and spatial analyses to examine the composite measure of SVI on COVID-19-related vaccination and mortality rates at the census tract level in Louisiana (Dong et al., 2021).

## Methodology

### Data

We retrieved the CDC SVI from the publicly available website (Agency for Toxic Substances & Disease Registry, 2018). We used census tract-level CDC SVI data for Louisiana from 2018. Summed percentile rankings at the census tract level for fifteen US census variables were used to calculate the overall SVI for each parish (shown in Table 1). For each parish, we counted the total number of adults who had been vaccinated. We used that number to explore the relationship between the CDC SVI and vaccination completion (who completed two doses of Pfizer COVID vaccine) rate. First, we downloaded the publicly available COVID-19 vaccination data for all adults who had completed two doses of the Pfizer vaccine from the Louisiana Department of Health website for all 64 parishes in Louisiana (Louisiana Department of Health, 2020) from December 2020 to October 2021. Each of the four parameters and the overall social vulnerability score was evaluated as a potential vulnerability indicator at the census tract level. We then merged the SVI data with the Louisiana Department of Health data for each census tract. Census tract-level rankings are a more precise measure of social vulnerability than parish-level rankings. Finally, we averaged the SVI values by the parish to provide a single SVI score representing each parish. The variables were selected based on four parameters: socioeconomic status, household composition, minority status and language, and housing and transportation. These parameters represent socioeconomic and demographic characteristics within a parish. The scores ranged from 0 to 1, with the highest level of social vulnerability described by 1 and the lowest level of social vulnerability represented by 0. The CDC SVI dataset provides census tract-level rankings for each variable, each parameter, and an overall ranking.

**Table 1:** Social Vulnerability Index: parameters and variables

SVI parameter	Variables included
Socio-economic status	% Below poverty
	% Unemployed
	% Income
	% No high school diploma
Household composition and disability	% Aged 17 years or younger
	% Aged 65 years or older
	% Single parent household
	% Civilian with disability
Minority status and language	% Minority
	% Aged 5 years or older; speaks English “less than well”
Housing and transportation	% Multi-unit structures
	% Mobile homes
	% Crowding (more people than rooms)
	% Households with no vehicle
	% Group quarters

### Data analysis

#### Spatial autocorrelation analysis

Spatial autocorrelation measures the similarity or dissimilarity of a variable with itself or other variables. Positive values indicate similarity between the variables, and negative values indicate dissimilarity between the variables. To examine the strength and significance of spatial effects, we used both univariate and bivariate Moran’s I test to assess spatial autocorrelation. Positive spatial autocorrelation (> 0) signifies the clustering of similar values, while negative spatial autocorrelation (< 0) indicates that different values are adjacent to one another. An output value of 0 illustrates complete spatial randomness. The further the value is from 0, the stronger the spatial autocorrelation (Devine et al., 1994; Atchade et al., 2011). We conducted a univariate global and local Moran’s I test for vaccination completion (who had completed two doses of the Pfizer vaccine), COVID-19-related mortality, and SVI using percentile rankings. We also used the Bivariate global and local Moran’s I test for vaccination completion, COVID-19-related mortality with overall percentile ranking score, and again with each of the four parameters. We also applied Empirical Bayesian Smoothing (EBS) to mortality rates and repeated the univariate and bivariate tests for spatial autocorrelation. EBS uses the mean number of deaths and population of a parish and its first-order neighbors to assign a new expected rate. The lower the rate, the more information the algorithm will draw from the neighboring parishes to smooth the rate. This is a method commonly used to stabilize rates in areas with small numbers, as is the case with mortality in this study (Devine et al., 1994; Atchade et al., 2011). We calculated a first-order queen’s contiguity spatial weights matrix and ran 999 Markov Chain repetitions for univariate and bivariate Moran’s I tests. The degree of spatial autocorrelation is assessed using the values from all first-order neighboring parishes to determine whether the area has a higher or lower mean. In addition, we conducted a geographically weighted Pearson correlation between COVID-19 mortality, vaccination rate, and overall SVI. Using a moving window approach, the weighted Pearson correlation examines whether the association between two variables varies geographically. We used R Software (R 3.3.1) for statistical analysis.

### Regression analysis

Due to the spatial nature of COVID-19-related data, we explored ordinary least square (OLS) models of COVID-19-related vaccination and mortality rates for any violations in uncorrelated error. COVID-19 corresponding mortality rates were log-transformed to fit a normal distribution. We defined a first-order queen’s contiguity spatial weights matrix in R. The OLS regression diagnostics were conducted for regressions, including the Lagrange Multiplier (LM) for spatial lag and Moran’s I residual autocorrelation. We examined the overall percentile rank per parish for potential associations with COVID-19 related vaccination and mortality rates for all regressions.

## Results

### Descriptive statistics

**Fig. 1:**
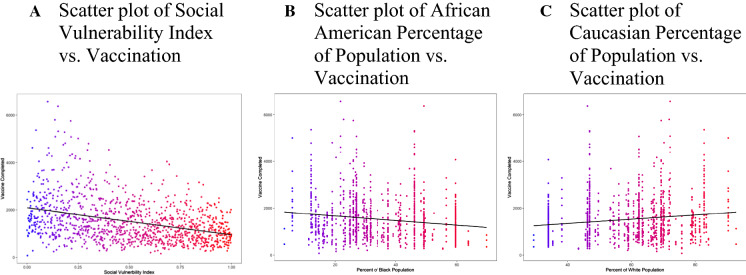
Analysis of the association between vaccination completion and SVI demonstrated a negative association between SVI and Vaccination rate (*p* < 0.001), which means that as the social vulnerability index increased, the vaccination rate decreased. The second and third plots show that the vaccination rate was lower where the African American percentage of the population was higher (*p* < 0.001) and higher where the Caucasian percentage of the population was higher (*p* < 0.001)

At the time of this study, COVID-19 vaccination completion rates (who had completed two doses of the Pfizer vaccine) in Louisiana were disproportionately lower among Blacks. This group represented over half of all mortality. Louisiana’s population consists of 32.8% Blacks and 62.8% whites (US Census Bureau, 2021). When evaluating vaccination rates, we found a positive correlation between the white population and a negative correlation for the black population. The relationship between vaccination and the percentage of the white and Black populations is shown in Fig. 1. The scatter plots of vaccination rates, and parameters are shown in supplementary Fig. 1A.

**Fig. 2:**
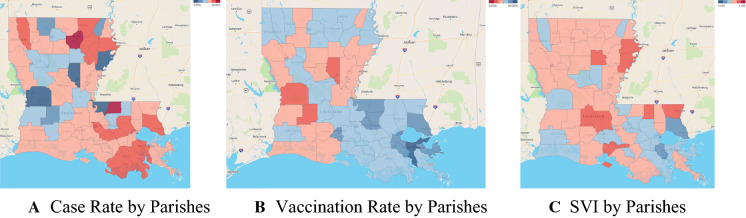
Distribution maps of COVID-related vaccination rates, mortality rates, and SVI ranking. The first two maps provide spatial context for COVID-related vaccination and mortality rates across Louisiana, and the third map gives spatial context to social vulnerability across Louisiana. Quartile breaks identify the higher rates in areas of higher social vulnerability

A geographical exploration of the vaccination rate, mortality rate, and social vulnerability index generally reveals low and higher mortality rates in areas of higher social vulnerability, presenting evidence for further spatial and statistical exploration. A spatial heatmap of case rate, vaccination rate, and social vulnerability index is shown in Fig. 2. All maps are visualized using quartile class breaks.

The vaccination and mortality rates are shown by region in Table 2. The four parameters are shown by region in supplementary Table 1A.

**Table 2:** Vaccination and mortality rates by region in Louisiana

Region	Population	Total deaths	Mortality rate (%)	Series completed	Vaccination rate (%)
1–New Orleans	915,823	2,148	0.23	438,067	49.00
2–Baton Rouge	697,665	1,753	0.25	257,047	37.74
3–South Central	410,304	1,151	0.28	130,400	32.38
4–Acadiana	622,211	1,573	0.25	182,778	30.16
5–Southwest	313,053	873	0.28	75,794	25.19
6–Central	308,882	1,021	0.33	77,580	25.42
7–Shreveport/Bossier	548,890	1,873	0.34	57,589	29.03
8–Monroe	362,749	1,255	0.35	100,967	28.59
9–Northshore	595,412	1,643	0.28	189,995	32.90

### Spatial autocorrelation

#### Univariate global Moran’s I

Results of the univariate global Moran’s I analysis revealed a significant positive spatial autocorrelation in overall rankings (0.38, *p* < 0.001), COVID-19 vaccination rates (0.56, *p* < 0.001), and COVID-19-related mortality rates (0.74, *p* < 0.001). However, significant clusters were observed for COVID-19-associated cases in different regions of Louisiana.

#### Bivariate global Moran’s I test

**Fig. 3:**
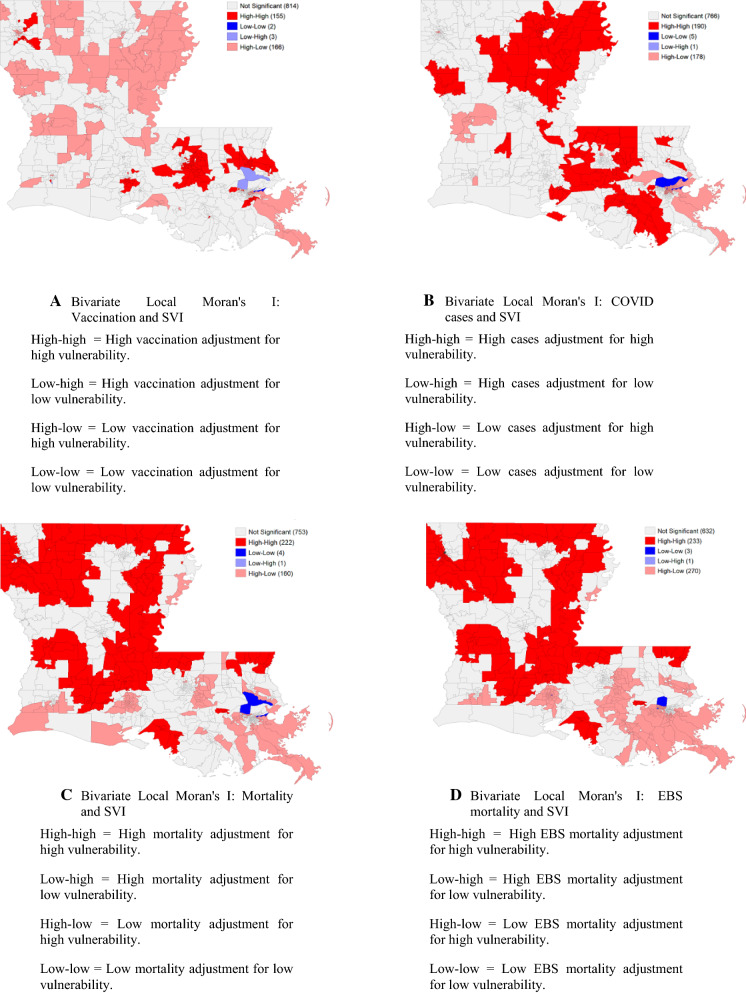
Cluster maps of COVID-related vaccination rates, mortality rates, and SVI ranking in Louisiana. **A** local hot spots for COVID-related vaccination and SVI values. **B** local hot spots for COVID-related cases and SVI values. **C** local hot areas for COVID-related mortality and SVI values. **D** local hot spots for EBS-smoothed COVID-related mortality and SVI values (α = 0.05)

Bivariate global Moran’s I analysis of COVID-19-related vaccination rates and overall CDC SVI rank demonstrated clustering of similar values (−0.21, *p* < 0.001), meaning that globally, areas with low vaccination rates tended to have high percentile SVI rankings. The bivariate Moran’s I analysis, COVID-19 related mortality rates, and overall CDC SVI rank showed a significant spatial relationship between mortality rates and ranks (0.15, *p* < 0.001). The bivariate Moran’s I smoothed rates of COVID-19 related mortality, and overall rank revealed clustering of similar values (0.15, *p* < 0.001), meaning that areas with high COVID-19 related mortality rates had high CDC SVI percentile ranks. Figure 3 displays the results of the bivariate Local Moran’s I analysis, which identifies significant local hot or cold spots. The bivariate Moran’s I for COVID cases, vaccination and mortality rates, and overall CDC SVI rank showed significant spatial relationships between mortality rates and SVI ranks (0.03, *p* < 0.01). Additionally, the bivariate Moran’s I that examined smoothed rates of extreme COVID-19-related mortality and overall SVI rank revealed clustering of similar values (0.02, *p* < 0.01), which demonstrated that areas with high COVID-19-related cases, low vaccination rates, and high mortality rates had high CDC SVI percentile ranks.

Breaking down the CDC SVI into its four parameters, we further examined the relationships between social vulnerability parameters and the COVID-19 vaccination separately and together (Table 3). Again, significant clustering of similar values was found with vaccination rates, mortality rates, and all parameters. Table 3 shows all bivariate analysis results for the vaccination rate, mortality rates with overall SVI, and each parameter.

**Table 3:** Results of bivariate global Moran's I analysis for COVID-related vaccination and mortality

Variables	Independent variable	Vaccination rate (*p*-value)	Mortality rate (*p*-value)	EBS mortality (*p*-value)
Parameter 1	Socio-economic status	0.042 (0.001)	0.034 (0.01)	0.021 (0.001)
Parameter 2	Household composition and disability	0.041 (0.001)	0.032 (0.01)	0.024 (0.001)
Parameter 3	Minority status and language	0.038 (0.001)	0.036 (0.01)	0.022 (0.001)
Parameter 4	Housing and transportation	0.047 (0.001)	0.031 (0.01)	0.021 (0.001)
Overall SVI	All 4 parameters	0.032 (0.001)	0.030 (0.01)	0.022 (0.001)

#### Geographically weighted Pearson correlation

There is variability in the geographically weighted Pearson correlation between COVID-19 related vaccination, mortality, and overall SVI by location. The geographically weighted Pearson correlations for vaccination rate were −0.35 (95% CI: −0.41 to −0.30). The geographically weighted Pearson correlation for the mortality rate was 0.15 (95% CI: 0.09–0.21). These findings’ large range of values indicated substantial geographic variation in the two relationships. This suggested that the relationship between SVI and COVID-19-related vaccination rates and mortality was inconsistent across all locations. This supports the interpretation and number of hot spots identified in the bivariate Local Moran’s I analysis shown in Fig. 3.

We used spatial lag regression to determine the association between SVI and COVID-19 related vaccination. Overall, an increase in the ranking was associated significantly with a decreased vaccination rate by the parish. For every 10% increase in the overall ranking, the vaccination rate decreased by 51%. All parameters had significant negative associations with vaccination. Household composition and disability, minority status and language, housing, and transportation (with a decrease of 60%) had the strongest association. We used spatial lag regression to determine the association between SVI and COVID-19 related mortality. Overall, increases in the ranking were significantly associated with a decrease in the vaccination rate by the parish. The mortality rate increased by 61% for the overall CDC SVI ranking. All parameters had significant positive associations with mortality.

## Discussion

Our findings demonstrated a significant association between SVI and lower COVID-19 vaccine uptake and higher mortality in Louisiana. A previous study carried out in Louisiana used *negative binomial regression with a random intercept to account for the possible spatial autocorrelation* and found that COVID-19 incidence was associated with SVI (Biggs et al., 2021) To our knowledge, this is the first geospatial study to investigate the spatial correlation between the social vulnerability score and vaccine uptake and mortality. Therefore, it is critical that the impact of social determinants of health on COVID-19 vaccination uptake and mortality are recognized and translated into outreach efforts and strategies to address inequities.

We found that vaccine uptake is low among Blacks and in socially vulnerable regions compared to uptake in whites and high-income areas. A high social vulnerability index suggests a population at risks, such as those with lower levels of income and education, limited healthcare access, housing density, transportation barriers, and inadequate health literacy. These social determinants of health influence people’s health choices and increase their health challenges (Tipirneni et al., 2021; Nayak et al., 2020). By evaluating social and cultural factors at the community level, public health, government officials, and healthcare professionals can more effectively tailor resources and communication to address health disparities, including vaccine uptake and mortality.

Our findings are consistent with those in the literature. For example, Kim et al. found that COVID-19 disproportionately affected poor and highly segregated Black communities in Chicago census tracts (Kim et al., 2020). They also reported spatial clusters of social vulnerability, with an associated increase in mortality rate. Other studies include those of Taylor et al., (2022), who showed the disproportionate effect of COVID-19 among Blacks; Gaynor et al. (2020), who used the SVI and found racial disparities in COVID-19 outcomes; and Scott et al. (2022), who used geographically weighted regression models and found racial and economic segregation in COVID-19 cases. In addition, other recent studies have found a positive association between COVID-19 incidence and mortality (Madhav et al., 2020; Nayak et al., 2020; Kim et al., 2020; Karaye et al., 2020; Selden et al., 2020), which agreed with our findings.

Our geospatial analysis helped pinpoint hot spots of COVID-19-related outcomes in Louisiana (Fig. 2). The spatial lag regression analysis further supported the spatial relationship between COVID-19-related outcomes and SVI. A recent study showed that SVI is better for targeting larger geographic areas compared to the Area Deprivation Index (ADI) (Hughes et al., 2021). In addition, SVI is better used to target specific neighborhoods with more significant social disadvantages. *Tipirneni *et al. (2021) showed that “ADI can be used as an alternative to SVI, but with the caveat that SVI is more useful for larger geographic areas because it focuses on the county level, while ADI is better for targeting smaller areas because it includes data at the level of neighborhoods but lacks data on race/ethnicity.” All our spatial and statistical tests of individual and overall parameters identified an association between COVID-19 vaccine uptake and the mortality rate.

Louisiana has been identified as a state with a higher SVI score, suggesting that during a public health emergency, it is more likely to require support before, during, and after the crisis. According to Acadiana’s News channel report, since the beginning of the pandemic, there has been a total of 755,631 cases of COVID-19 and 14,462 COVID-19-related deaths up to and including November 2020 (Louisiana Department of Health, 2020, Louisiana Office of the Governor, 2021). Before the fourth pandemic wave (August-October 2021), Louisiana had one of the lowest vaccination rates in the nation (30%); more than 80% of new cases, hospitalizations, and deaths were in unvaccinated patients. Application of the SVI in this setting to identify states like Louisiana could have helped provide public health resources, outreach strategies, and communication and preventive strategies.

This study has limitations. First, vaccination was introduced in different tiers based on population risk factors (Louisiana Office of the Governor, 2021). Future studies may consider sub-group analysis based on when the vaccine was available to each group. Second, the region with the highest mortality does not have the lowest vaccination rate, demonstrating this issue’s complexity, which is compounded by a lack of access to health care, possible lack of clinical expertise, and access to advanced therapies such as extracorporeal membrane oxygenation.

## Conclusion

This study demonstrates the reliability of a geo-marker tool used to identify at-risk populations. We showed that areas with higher SVI rankings experienced a more significant impact from COVID-19. This continues to be a problem in the state, as disparities in vaccination coverage have increased over time, particularly between regions with low versus high social vulnerability. Ensuring equitable access to COVID-19 vaccination, testing, and treatment facilities are crucial, as demonstrated by the geographical linkage between COVID-19 vaccine uptake and social vulnerability. We present evidence of the utility of the CDC’s GRASP database and SVI tool in identifying areas with high social disparity and low vaccination in Louisiana. In the future, communities, health systems, public health, and government officials should consider using this tool to identify at-risk areas and dedicate public efforts and resources to overcoming barriers that increase health disparities, as has occurred with lower COVID-19 vaccination and higher mortality rates.

## Data Availability

The datasets for this study can be found in the Louisiana Department of Health.
